# COVID-19 Impact on Lipid Profile and Metabolome in Patients with Type 2 Diabetes Mellitus: Mini-Review

**DOI:** 10.2174/0115733998404775251202121401

**Published:** 2025-02-13

**Authors:** Karolina Pawłuszkiewicz, Gabriela Augustynowicz, Emil Paluch, Barbara Porębska, Zofia Bielenin, Anita Maria Faltus, Katarzyna Madziarska, Joanna Kluz, Agnieszka Bronowicka-Szydełko

**Affiliations:** 1 Faculty of Medicine, Wroclaw Medical University, Wroclaw Medical University, Wroclaw, Poland;; 2 Department of Biochemistry and Immunochemistry, Wroclaw, Poland;; 3 Clinical Department of Diabetology, Hypertension and Internal Diseases, Institute of Internal Diseases, Wroclaw Medical University, Wroclaw, Poland;; 4 Department of Microbiology, Wroclaw Medical University, Wroclaw, Poland;; 5 Department of Emergency Medicine, Wroclaw Medical University, Wroclaw, Poland

**Keywords:** COVID-19, diabetes mellitus, insulin resistance, metabolome, lipid metabolism, T2DM2 patients

## Abstract

**Aim:**

This review aims to explore the pathophysiological mechanisms by which SARS-CoV-2 affects lipid metabolism and the metabolome in patients with Type 2 Diabetes Mellitus (T2DM). It compares these alterations with those observed in non-diabetic individuals and proposes strategies to mitigate potential long-term metabolic complications.

**Methods:**

A narrative review was conducted using PubMed, Scopus, and Google Scholar to identify studies published between 2004 and 2025 concerning adult T2DM patients during or after COVID-19 infection. The analysis focused on lipid profiles, including total cholesterol, high-density lipoprotein (HDL), low-density lipoprotein (LDL), and triglycerides, along with hemoglobin A1C levels and COVID-19 severity. Search terms included: (Post-COVID-19) AND (Diabetes Mellitus Type 2), ((Post-COVID-19) AND (Diabetes Mellitus Type 2)) AND (Lipid Profile), ((COVID-19) AND (Lipid Profile)) AND (Diabetes Mellitus Type 2), (Post-COVID-19) AND (Lipid Metabolism), (COVID-19) AND (Lipid Metabolism), (Post-COVID-19) AND (Metabolome), (COVID-19) AND (Metabolome).

**Results:**

Post-COVID-19, patients with T2DM exhibit persistent disturbances in lipid metabolism and broader metabolic pathways. SARS-CoV-2 infection amplifies metabolic dysregulation, leading to decreased levels of total cholesterol, HDL, and LDL, while triglyceride levels remain variable. Mechanistically, the virus disrupts lipid homeostasis by upregulating genes such as CD36 and peroxisome proliferator-activated receptor gamma (PPAR-γ) and by altering amino acid metabolism, particularly within the tryptophan-kynurenine and glutamine pathways.

**Discussion:**

Enhanced oxidative stress and lipid peroxidation contribute to systemic inflammation and endothelial dysfunction. Furthermore, glycemic control worsens due to increased insulin resistance and pancreatic interactions involving angiotensin-converting enzyme 2 (ACE2).

**Conclusion:**

COVID-19 significantly alters lipid metabolism and the metabolomic profile in individuals with T2DM. Longitudinal studies are warranted to clarify the duration and clinical impact of these changes and to develop personalized therapeutic strategies for this high-risk population.

## 1 INTRODUCTION

A new form of pneumonia caused by coronavirus was reported in December 2019 in Wuhan, China [1]. The International Committee on Taxonomy of Viruses identified and named this virus severe acute respiratory syndrome coronavirus 2 (SARS-CoV-2). The new name for the disease caused by SARS-CoV-2, CoV-disease (COVID-19), was announced on February 11th, 2020, by the World Health Organization (WHO). On March 11th, 2020, COVID-19 was declared a pandemic [2]. As of 15 June 2025, SARS-CoV-2 infection was reported in more than 778 million people and has caused 7.097 million deaths [3]. SARS-CoV-2 belongs to the genus Betacoronavirus (Beta-CoV) and is a member of the Coronaviridae family [4]. Its genome organization closely resembles that of other coronaviruses, comprising open reading frames, with approximately 67% of the genome encoding nonstructural proteins and 33% encoding glycoproteins and structural proteins. Fig. (**1**) presents four key structural proteins, spike (S), membrane (M), envelope (E), and nucleocapsid (N), which are essential for viral replication and maintenance [5].

SARS-CoV-2 spreads primarily through direct contact with infected individuals or *via* respiratory droplets. Upon inhalation, the virus targets epithelial cells, where it replicates and subsequently enters the bloodstream. SARS-CoV-2 exhibits a stronger binding affinity to the angiotensin-converting enzyme 2 (ACE2) receptor compared to other coronaviruses. These receptors are widely expressed in various tissues, including the nasal and oral mucosa, lungs, heart, liver, and gastrointestinal tract [6]. Moreover, many of these tissues contain another key protein that influences the intensification of viral replication and spread, Transmembrane Serine Protease 2 (TMPRSS2), a protein facilitating viral fusion [7]. The systemic dissemination of the virus enables infection of multiple ACE2- and TMPRSS2-expressing organs, leading to extrapulmonary manifestations such as gastroenteritis, hypertension, tachycardia, insomnia, headaches, taste disorders, and skin infections [8]. Risk factors associated with severe COVID-19 and mortality include advanced age and comorbidities such as cardiovascular diseases, chronic lung disease, malignancies, immunosuppression, chronic kidney disease, and diabetes [9].

A subset of individuals experiences persistent, multisystemic symptoms following acute SARS-CoV-2 infection, a condition collectively termed long COVID, also known as Post-Acute Sequelae of COVID-19 (PASC), Post-Acute COVID-19 Syndrome (PACS), or Post-COVID Conditions (PCCs) [10, 11]. Table **1** outlines the temporal classification of COVID-19 illness as defined by the National Institute for Health and Care Excellence (NICE, 2024), distinguishing between disease phases based on symptom duration [12].

In 2024, both the World Health Organization (WHO) and the National Academies of Sciences, Engineering, and Medicine (NASEM) proposed distinct definitions of long COVID. The WHO defines it as: “Post COVID-19 condition is characterized by a range of symptoms that usually start within 3 months of the initial COVID-19 illness and last at least 2 months.” In contrast, NASEM describes long COVID as: “Long COVID is an infection-associated chronic condition that occurs after SARS-CoV-2 infection and is present for at least 3 months as a continuous, relapsing and remitting, or progressive disease state that affects one or more organ systems” [13, 14]. These definitions emphasize the emergence of new or persistent symptoms following confirmed or probable COVID-19 infection, typically featuring a heterogeneous symptom profile. Common clinical presentations include dyspnea, cognitive impairment (“brain fog”), dysautonomia, and fatigue, though more than 200 distinct symptoms have been documented across systems [15].

Type 2 Diabetes Mellitus (T2DM) is characterized by chronic hyperglycemia, insulin resistance, and a relative deficiency of insulin. Persistent hyperglycemia and systemic inflammation impair the immune response, rendering individuals with T2DM more vulnerable to complications, particularly from viral infections [16]. Historically, individuals with T2DM have exhibited increased morbidity and mortality during viral pandemics. For example, during the 2002-2003 SARS-CoV-1 outbreak, patients with T2DM had elevated risks of acute complications and mortality [17]. Similarly, during the 2009 influenza A (H1N1) pandemic, T2DM was associated with a fourfold increased likelihood of intensive care unit (ICU) admission [18]. Furthermore, during the 2012 Middle East Respiratory Syndrome Coronavirus (MERS-CoV) outbreak, individuals with T2DM experienced a 35% higher mortality rate than the general population [19]. Patients with T2DM are more likely to experience severe COVID-19 symptoms and require advanced medical interventions, including mechanical ventilation. Uncontrolled T2DM exacerbates the risk of poor outcomes. Independent predictors of mortality in these patients include a known history of diabetes and fasting glucose levels ≥ 7.0 mmol/L before steroid treatment [20].

This review aims to elucidate the potential pathophysiological mechanisms by which SARS-CoV-2 infection has a substantial impact on the lipid profile and metabolome in patients with T2DM and to describe its clinical consequences by comparing metabolic changes in patients with and without T2DM.

## 2 METHODS

This narrative review has been conducted using Google Scholar, PubMed, and Scopus, primarily incorporating studies published between 2020 and 2025, with occasional reference to earlier seminal works pertinent to the topics discussed. Articles were selected based on predefined criteria: studies focusing on adult patients with T2DM during or post-COVID-19 pandemic, reporting lipid profiles (total cholesterol (TC), high-density cholesterol (HDL), low-density cholesterol (LDL), triglycerides (TG) levels) and hemoglobin A1C (HbA1c) levels, as well as COVID-19 severity. Excluded were studies on type 1 diabetes, pediatric populations, new-onset diabetes post-COVID-19, and those lacking lipid profile data. Key search terms included: (Post-Covid-19) AND (Diabetes mellitus type 2); ((Post-Covid-19) AND (Diabetes mellitus type 2)) AND (Lipid profile); ((Covid-19) AND (Lipid profile)) AND (Diabetes mellitus type 2); (Post-Covid-19) AND (Lipid metabolism); (Covid-19) AND (Lipid metabolism); (Post-Covid-19) AND (Metabolome); (Covid-19) AND (Metabolome).

## 3 PATHOGENESIS OF THE IMPACT OF COVID-19 ON LIPID PROFILE AND METABOLOME

### 3.1 Inflammation and Hyperglycemia in COVID-19

The proinflammatory state during COVID-19 infection plays an active role in the pathogenesis of uncontrolled hyperglycemia, insulin resistance, and the development of chronic complications in patients with T2DM [21]. Moreover, even patients without T2DM who experience prolonged hyperglycemia are at increased risk of a more severe disease course and subsequent complications [22].

Changes in the immune system, including alterations in populations of specific cytokines and chemokines, shifts in the number and activation state of various leukocyte populations, and increased apoptosis and tissue fibrosis, further exacerbate the inflammatory state in those patients. Compared to patients without diabetes, individuals with T2DM exhibited higher levels of interleukin 6 (IL-6), ferritin, high-sensitivity C-reactive protein (hsCRP), and d-dimer--predictors of a cytokine storm and a hypercoagulable state, leading to rapid clinical deterioration [21]. Uncontrolled hyperglycemia may influence ACE2 levels and COVID-19 severity by altering glycosylation of ACE2 and the viral spike protein, potentially enhancing viral binding and modulating the immune response [22]. On the other hand, the release of stress hormones and the inflammatory state can cause stress hyperglycemia. Moreover, insulin resistance develops as a result of a cytokine storm triggered by an inflammation [23]. All those changes worsen the prognosis [21]. Procalcitonin has also proven to be a significant diagnostic marker for COVID-19. This marker of bacterial superinfection serves as a key predictor of severe COVID-19, highlighting the link between metabolic dysregulation and inflammation. Its monitoring is vital for risk assessment and management, especially in individuals with diabetes or insulin resistance. Optimizing glucose control is essential to improve outcomes, minimize secondary infections and renal dysfunction, and reduce ICU stays and ventilator dependency [21].

### 3.2 Metabolic Side Effects of COVID-19 (Renin-angiotensin-aldosterone System, Oxidative Stress, and Advanced Lipid Peroxidation end Products)

COVID-19 infection affects the renin-angiotensin-aldosterone system (RAAS) by binding to ACE2 receptors, which are crucial for maintaining blood pressure and glucose homeostasis. Fig. (**2**) illustrates the effects of ACE2 inhibition by SARS-CoV-2 on inflammatory pathways, pancreatic β-cell function, and the development of insulin resistance, based on data from previous studies [22, 23].

The spike protein of SARS-CoV-2 binds with high affinity to ACE2, facilitating viral entry into cells, which exacerbates inflammation. ACE2 is the primary enzyme in the RAAS, whose role is to regulate water and sodium balance, vascular resistance, and thus blood pressure. On this account, strategies targeting ACE2 activity in COVID-19 could offer therapeutic benefits [22, 23].

Oxidative stress (OS) plays a critical role in T2DM and its complications, as well as in the progression of COVID-19 in diabetic patients. Reactive oxygen species (ROS), primarily produced in mitochondria, are central to OS. In diabetes, excess ROS results from mitochondrial dysfunction, hyperglycemia-driven metabolic pathways (*e.g.*, advanced glycation, polyol pathways, lipid peroxidation), and chronic inflammation. This imbalance damages proteins, lipids, and DNA, impairing insulin sensitivity, β-cell function, and vascular health [24]. OS contributes to microvascular (*e.g.*, nephropathy, retinopathy) and macrovascular (*e.g.*, atherosclerosis, myocardial infarction) complications in T2DM.

The interaction of SARS-CoV-2 with the ACE2 receptor disrupts cellular signaling pathways, primarily through the activation of the nuclear factor kappa-light-chain-enhancer of activated B cells (NF-κB). This leads to increased production of pro-inflammatory cytokines (TNF-α, IL-6), which intensifies vascular damage and promotes both local and systemic inflammation. SARS-CoV-2 infection exacerbates OS by inducing a cytokine storm, endothelial dysfunction, and further ROS production. Elevated glucose levels in diabetic COVID-19 patients further stimulate cytokine activity, perpetuating a vicious cycle of inflammation and organ damage [24].

Advanced lipid peroxidation end products (ALEs), such as 4-hydroxynonenal (4-HNE) and trans-2-nonenal, are toxic aldehydes generated during lipid peroxidation. Their levels are elevated in conditions associated with increased oxidative stress, characteristic of both T2DM and SARS-CoV-2 infection [25, 26]. In diabetes, chronic hyperglycemia exacerbates OS, leading to enhanced lipid peroxidation and elevated levels of ALEs, including 4-HNE and trans-2-nonenal. Among these, 4-HNE is particularly reactive, forming adducts with DNA, proteins, and phospholipids, which disrupt cellular functions. Research suggests that 4-HNE can impair DNA repair mechanisms by interacting with repair proteins, thereby contributing to the development of diabetes-related complications [25].

Similarly, SARS-CoV-2 infection induces intense inflammation and OS, increasing lipid peroxidation and the production of ALEs [27]. Although specific data on 4-HNE and trans-2-nonenal levels in post-COVID-19 patients are limited, OS associated with the infection likely leads to elevated amounts of these aldehydes [26]. Such elevations may contribute to cellular damage and the development of long-term complications [28].

In summary, both T2DM and SARS-CoV-2 infection are linked to increased OS and lipid peroxidation, leading to elevated levels of ALEs, which contribute to cellular injury and the development of complications in these conditions [26, 28].

### 3.3 Impact of COVID-19 on Lipid Profile and Hemoglobin A1C (HbA1c) Levels in Patients With and Without T2DM

SARS-CoV-2 infection significantly alters the lipid profile in both individuals with and without T2DM, affecting patients during the acute phase and post-infection. Dyslipidemia is commonly observed in COVID-19 patients and manifests in various patterns depending on the presence of metabolic comorbidities, disease severity, and follow-up duration [29].

In non-diabetic individuals, the most consistently reported changes include reductions in total cholesterol (TC) and high-density lipoprotein (HDL) levels, while low-density lipoprotein (LDL) and triglyceride (TG) levels remain relatively stable or show mild variability [29, 30]. In a retrospective study by Zhao *et al.*, which included healthy individuals, COVID-19 patients, and those with chronic obstructive pulmonary disease (COPD), it was shown that COVID-19 patients had significantly lower levels of TC (*p* < 0.001) and HDL (*p* < 0.001) compared to healthy controls [30]. Similar findings in COPD patients suggest a shared mechanism driven by systemic inflammation.

The loss of HDL and reduced TC levels during COVID-19 may have serious clinical implications, as HDL is known to modulate immune responses and protect cardiovascular health. Lower lipid levels may impair the body's capacity to control inflammation and increase the risk of cardiovascular complications [31]. Increased TG levels during acute infection are attributed to enhanced very low-density lipoprotein (VLDL) production, resulting in the formation of small, dense LDL particles, which are more atherogenic. Additionally, secreted phospholipase A2 (sPLA2) activity increases during infection, hydrolyzing HDL phospholipids and promoting LDL oxidation, which further drives atherosclerosis [31].

Lipid disturbances may persist during convalescence, especially in patients with post-COVID syndrome. Therefore, routine lipid monitoring in COVID-19 survivors is recommended to detect early cardiovascular risks and initiate preventive interventions [29, 32].

In patients with T2DM, lipid disturbances are often more pronounced. A positive correlation has been documented between HbA1c levels and LDL, VLDL, and TG, but not with HDL [33]. The COVID-19 pandemic has had a detrimental impact on the lipid profile of T2DM patients from the early stages. Retrospective and cross-sectional studies reported significant reductions in HDL, LDL, and TC, particularly in critically ill patients [34]. Notably, a negative correlation was observed between lipid changes and hospitalization length [34].

No significant differences were found between insulin-treated patients and those on oral antidiabetic drugs, indicating that lipid alterations are likely due to the infection itself rather than the treatment modality [35]. One proposed explanation for LDL reduction was impaired liver function, but more compelling evidence supports the direct impact of SARS-CoV-2 on apolipoproteins and gene expression regulating lipid metabolism, including CD36 and PPAR-γ [36, 37].

Moreover, COVID-19 is associated with hyperglycemia, both in individuals with and without diabetes. The virus may bind to ACE2 receptors on pancreatic β-cells, impairing insulin secretion. The inflammatory state and elevated levels of proinflammatory cytokines (*e.g.*, IL-6, TNF-α) further exacerbate insulin resistance [22-24].

In the Egyptian study, a significant increase in HbA1c was observed in T2DM patients after COVID-19 infection - from 7.48% ± 0.94% to 8.52% ± 1.17% within 12 months (*p* < 0.001) [38]. Other studies have demonstrated worsening glycemic control during the pandemic, even among patients who did not contract COVID-19. This deterioration was attributed to lockdowns, reduced access to medical care, increased stress, and decreased physical activity [39, 40]. Meta-analyses revealed significantly lower TC, LDL, and TG levels in retrospective studies, but these differences were not statistically significant in prospective studies (*p* > 0.05) [39].

Interestingly, regional variation has been noted. Studies from Japan and Korea did not observe significant changes in HbA1c levels among T2DM patients, suggesting that lifestyle and socioeconomic factors may modulate the metabolic response to the pandemic. Conversely, European studies confirmed both deterioration in glycemic control and characteristic lipid profile changes [41].

It is crucial to emphasize that hypolipidemia is not a benign state. It may reflect an intensified inflammatory response and has been associated with increased levels of inflammatory markers (IL-6, CRP, d-dimer) and worse COVID-19 outcomes [29, 32]. This supports the hypothesis that lipid profile changes in COVID-19 are not merely incidental but may serve as clinical indicators of disease severity and systemic inflammation.

### 3.4 Variability in Triglyceride Levels in COVID-19 Studies - Potential Sources of Discrepancy

Triglyceride (TG) levels in COVID-19 patients vary significantly depending on population characteristics, disease stage, and study methodology. Some studies report elevated TG during infection, while others show no significant changes or even reductions. Table **2** presents the main potential contributors to this variability [34, 39, 41-43].

These findings highlight the need for cautious interpretation of TG-related data. Future studies should aim to improve standardization and control for confounding variables when investigating metabolic changes in COVID-19.

### 3.5 Hypolipidemia as an Inflammatory Marker in COVID-19

The decrease in total cholesterol (TC), LDL, and HDL levels observed in COVID-19, particularly in patients with severe disease, may reflect not only metabolic dysregulation but also a robust inflammatory response. Observational studies have shown strong correlations between low lipid levels and elevated inflammatory markers such as interleukin 6 (IL-6), C-reactive protein (CRP), and d-dimer [29, 44]. The mechanisms underlying hypolipidemia involve the direct impact of inflammatory cytokines on lipid biosynthesis pathways and accelerated lipoprotein catabolism. IL-6 has been shown to inhibit lipoprotein lipase (LPL), impairing lipid uptake by peripheral tissues. Additionally, activation of the NF-κB pathway and altered expression of lipid receptors (*e.g.*, CD36, SR-B1) contribute to changes in HDL and LDL metabolism [45, 46]. Hypolipidemia may thus serve as a biomarker of severe COVID-19 and has been associated with poor outcomes. A meta-analysis involving over 2500 patients demonstrated that low LDL levels were associated with more than double the risk of mortality [29]. Therefore, lipid profile assessment in COVID-19 may have not only diagnostic but also prognostic value, particularly in patients with comorbidities.

### 3.6 Impact of COVID-19 on the Metabolome in Patients with and without T2DM

In patients with and without T2DM, SARS-CoV-2 infection has been shown to alter lipid and protein metabolism. To facilitate its replication, the virus upregulates genes associated with lipid metabolism, such as CD36, PPAR-γ, SREBP-1, and diacylglycerol acyltransferase-1 in monocytes, which enhance fatty acid oxidation [36, 37]. The identification of specific fatty acids, glycerophospholipids, and sphingolipids, including butyric acid and L-phenylalanine, which play a critical role in the inflammatory response, enables the prediction of COVID-19 severity [36, 47].

2-Hydroxybutyric acid, closely associated with lipid and carbohydrate metabolism, is elevated in critically ill patients with lactic acidosis and ketoacidosis [36, 48]. Studies have shown that patients with severe COVID-19 and T2DM have diminished levels of triacylglycerols containing C22:5 (DPA) and C22:6 (DHA) fatty acids. These changes may be linked to an improved inflammatory response, as n-3 fatty acids have well-known anti-inflammatory effects in immune cells [49, 50].

This hyperinflammatory state of severe COVID-19, at least partially, may be attributed to glutamine deficiency and impaired glutaminolysis that inhibits the polarization of M2 macrophages. These macrophages are a source of potent lipid metabolites that regulate the magnitude and duration of acute inflammation. Evidence suggests that L-glutamine levels significantly differ between the acute and convalescent phases of severe COVID-19 in paired serum samples [51, 52].

Hyaluronic acid (HA), a glycosaminoglycan component of the extracellular matrix (ECM), plays a pivotal role in water homeostasis, cell-matrix signaling, inflammation, angiogenesis, and cell migration. Severe COVID-19 symptoms are characterized by their deregulated production in the lungs, resulting in a “hyaluronic storm” [50], potentially linked to low glutamine levels and hyperglycemia in T2DM patients. A glutamine deficiency reduces the activity of glutamine-fructose-6-phosphate transaminase (GFAT), a rate-limiting enzyme in the hexosamine biosynthetic pathway (HBP), which is critical for HA synthesis, thereby exacerbating HA overproduction. Furthermore, hyperglycemia facilitates glucose flux through the uronic acid and HBP pathways, generating substrates for HA synthesis [53].

A significant reduction in L-phenylalanine and tyrosine levels during the convalescent phase of COVID-19 compared to the acute phase has also been observed. In sepsis and HIV-1 infection, elevated serum phenylalanine levels are associated with immune system activation and an increased risk of cardiovascular events [54-56].

Dysregulation in three metabolites within the tryptophan-kynurenine pathway--reduced L-tryptophan levels alongside increased kynurenine and 3-hydroxy-DL-kynurenine levels--further confirms the pathway's relevance in severe COVID-19 cases [57]. *in vitro*, tryptophan inhibits T- cell proliferation and sensitizes them to apoptosis, while the pathway itself modulates macrophage responses by targeting NAD+ coenzyme synthesis [58].

Reduced citrulline levels, an amino acid involved in arginine metabolism in the urea cycle, have been observed in patients with severe sepsis and acute respiratory distress syndrome (ARDS), mirroring changes noted in COVID-19 patients' blood samples. The metabolism of arginine to creatine and subsequently to creatinine may be associated with renal dysfunction in COVID-19 patients [51, 59].

Given the limited understanding of the precise impact of this infection on lipid, protein, and carbohydrate metabolism, further research is essential to uncover its underlying mechanisms and consequences, especially in patients with T2DM.

### 3.7 Regional Differences in Metabolic Consequences of COVID-19

Studies indicate that the metabolic impact of COVID-19-including dyslipidemia, glycemic control, and the risk of developing long COVID syndrome (LCS)-varies significantly across geographic regions, particularly between Asian and European populations. In countries such as Japan and South Korea, no significant changes in HbA1c levels were observed in patients with T2DM during the pandemic, possibly reflecting protective lifestyle factors, diet, and healthcare access [41]. Studies from South Korea and Japan reported mean differences in HbA1c of 0.02% (*p* = 0.84) and 0.05% (*p* = 0.66), respectively, indicating stable glycemic control [41]. In contrast, European studies documented worsening glycemic control along with marked reductions in TC, HDL, and LDL levels [41]. Data from China also revealed significant reductions in TC, HDL, and LDL among patients with severe COVID-19 [7,8], consistent with retrospective findings from Europe [41].

Interestingly, emerging data from post-COVID studies suggest an opposite long-term trend: increased total cholesterol (TC) and triglyceride (TG) levels in patients experiencing symptoms of long COVID syndrome (LCS) [43]. A meta-analysis of global studies reported that TG levels tended to be higher in more severe COVID-19 cases, although with considerable heterogeneity (standardized mean difference, SMD = +0.110, 95% confidence interval, CI [0.004-0.217], *p* = 0.042, I^2^ = 50.7%) [42]. This variability may be explained by regional differences in diet, physical activity, metabolic phenotype, and healthcare access.

A large prospective cohort study conducted in Germany with over 50,000 participants identified dyslipidemia (odds ratio, OR = 1.46, 95% CI [1.28-1.^65^], *p* < 0.001) and obesity (OR = 1.25, 95% CI [1.08-1.^44^], *p* = 0.003) as independent risk factors for developing LCS [60]. Table **3** presents a comparison of lipid and glycemic alterations observed in COVID-19 patients across cohorts from Asia (China, Japan, and Korea) and Europe, highlighting regional differences in metabolic responses to the infection [7, 8, 41-43, 60].

## 4 DISCUSSION

This review explores how changes in the lipid profile during COVID-19 affect human health, with correlations to disease severity and mortality. Table **4** provides an overview of the metabolic alterations associated with COVID-19 [22-29].

Findings on TG levels vary across studies, partly due to differences in study design and patient populations. Some reports lack appropriate control groups or rely on observational data, limiting their reliability [21,54].

Evidence suggests that SARS-CoV-2 infection has long-term metabolic consequences. A systematic review found that some patients develop newly diagnosed dyslipidemia, characterized by elevated triglycerides, LDL, and total cholesterol months after the infection, indicating the need for ongoing lipid monitoring [61]. Emiroglu *et al.* further linked obesity and elevated triglycerides post-infection with an increased risk of developing diabetes, supporting the predictive value of lipid profiling in assessing metabolic risk [62]. A cohort study of 228,266 individuals in Naples by Trimarco *et al.* reported a significant rise in dyslipidemia incidence during the pandemic (OR = 1.29, 95% CI [1.19-1.^39^]), confirming SARS-CoV-2 as a risk factor for lipid disturbances [63]. Similarly, the CovILD study (Sonnweber *et al.*), which followed 145 post-COVID-19 patients for 360 days, revealed that coexisting dyslipidemia and dysglycemia were associated with more severe disease progression and slower recovery [55, 64]. SARS-CoV-2 infection can lead to newly diagnosed lipid abnormalities, including elevated triglycerides, LDL, and total cholesterol, even months or years after recovery, suggesting that long-term lipid monitoring is essential in post-COVID-19 care, especially for patients with existing metabolic risk, such as T2DM [51, 61, 65, 66]. Elevated post-infection triglycerides and obesity have been linked to the later development of diabetes, indicating that monitoring lipid profiles could help predict and manage future metabolic risk in post-COVID-19 patients [62]. Increased incidence of dyslipidemia during the pandemic (OR = 1.29) further supports the notion that COVID-19 is a metabolic risk factor, justifying routine lipid monitoring in at-risk populations, including those with T2DM [63].

Dyslipidemia and dysglycemia were also associated with slower recovery and worse outcomes, reinforcing the need for integrated lipid and glucose monitoring in post- COVID-19 care for diabetic patients [64, 66]. Profound disruptions in lipid and amino acid pathways during infection suggest that long-term metabolic monitoring is critical in recovery management [67]. Regular monitoring of lipid parameters in diabetic patients after COVID-19 enables early detection of worsening insulin resistance and the risk of vascular complications [63-69].

Most journals report significant changes in lipid levels within one month of hospitalization, although some also present alterations 3-4 months later. Regardless of the duration, the use of medications is recommended [68]. Current evidence suggests statins as the first-choice therapy for post- COVID dyslipidemia. Atorvastatin and simvastatin appear to be the safest options for reducing LDL levels. To stabilize triglyceride and HDL levels, fibrates are highly recommended. In severe cases that do not respond to statins, PCSK9 inhibitors and niacin may be considered. Patients should also be regularly monitored by specialists and adhere to a low-fat diet combined with exercise [69, 70].

Rodriguez-Vera *et al.* highlighted the potential role of omega-3 supplementation in patients who developed dyslipidemia as a complication of COVID-19, suggesting possible therapeutic benefits [71]. After a minimum dose of 1 g of EPA per day, the researchers observed a significant increase in HDL, correlated with decreases in LDL/HDL and TC/HDL ratios. Significant reductions in LDL, VLDL, and TG were also reported, suggesting cardioprotective effects. Better results were potentially achieved in patients with baseline high LDL and VLDL. However, omega-3 is not yet a standard recommendation, and its mechanisms of action are not fully understood [71].

The virus-induced dysregulation of lipid metabolism genes, including CD36, PPAR-γ, and SREBP-1, alongside changes in pathways such as the tryptophan-kynurenine axis, underscores the need for further exploration [36, 37, 57]. Additionally, the role of glutamine in severe COVID-19 cases is notable, as reduced levels correlate with impaired metabolic responses and an enhanced inflammatory state [51, 52]. Dysregulated production of hyaluronic acid (HA) observed in severe lung involvement is linked to low glutamine levels and hyperglycemia in T2DM, further exacerbating inflammation and disease progression [50, 53]. Comprehensive studies focusing on these metabolic alterations, especially in T2DM patients, are critical for understanding pathophysiological mechanisms and guiding therapeutic strategies.

Practical recommendations for patient care with DM2

- Monitoring and strict control of glucose (including HbA1c), lipids, blood pressure, and inflammation during the infection and after it as part of cardiovascular and metabolic risk stratification [ 72 ].
- Well-adjusted therapy-antihypertensive, lipid-lowering, and/or insulin to prevent cardiometabolic consequences [59, 72].
- Follow-up examinations in the long term after COVID-19 infection [73-77].
- Diet and nutritional status modification to improve metabolism in patients with obesity [ 68 ].
- Exercise and Rehabilitation: Physical activity supports metabolic recovery post-infection [ 68 ].
- Individualized Treatment: Tailored approaches are essential due to patient variability.

## CONCLUSION

The COVID-19 pandemic has disproportionately affected individuals with T2DM, with oxidative stress (OS) emerging as a pivotal link between the two conditions. Increased levels of reactive oxygen species (ROS) and advanced lipid peroxidation products, such as 4-hydroxynonenal (4-HNE), contribute to systemic inflammation, endothelial dysfunction, and multiorgan injury. Patients with T2DM demonstrate elevated pro-inflammatory markers, including IL-6 and hsCRP, predisposing them to a cytokine storm and severe disease progression. SARS-CoV-2-mediated dysregulation of the renin-angiotensin-aldosterone system (RAAS) *via* ACE2 receptor binding exacerbates metabolic disturbances, including impaired glycemic control and lipid dysregulation. Notably, alterations in lipid profiles-characterized by reductions in total cholesterol, HDL-C, LDL-C, and ApoA1-are frequently observed, while triglyceride levels may rise in severe inflammatory states.

Effective management requires a precision-medicine approach encompassing tight glycemic control (*e.g.*, metformin), lipid-lowering therapy (*e.g.*, statins), antihypertensives, and lifestyle interventions targeting obesity and inflammation. Practical strategies should include continuous monitoring of glucose, lipids, blood pressure, and inflammatory markers; individualized pharmacotherapy; structured long-term follow-up; dietary optimization; and post-infection rehabilitation to restore cardiometabolic homeostasis.

## Figures and Tables

**Fig. (1):**
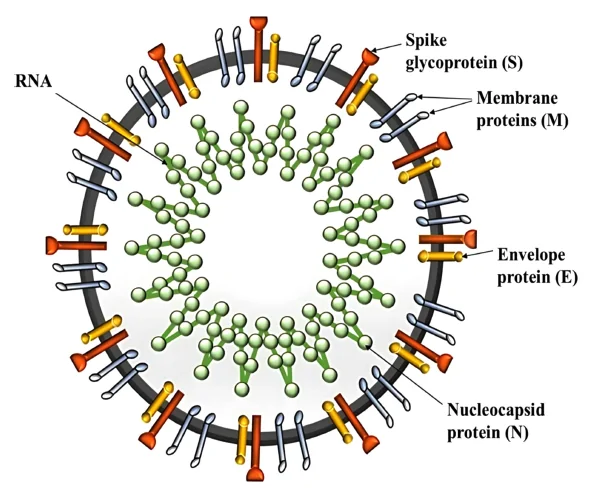
The structure of severe acute respiratory syndrome coronavirus 2 (SARS-CoV-2) [5]. RNA, Ribonucleic acid; N, Nucleocapsid protein; E, Envelope protein; M, Membrane proteins; S, Spike glycoprotein.

**Fig. (2):**
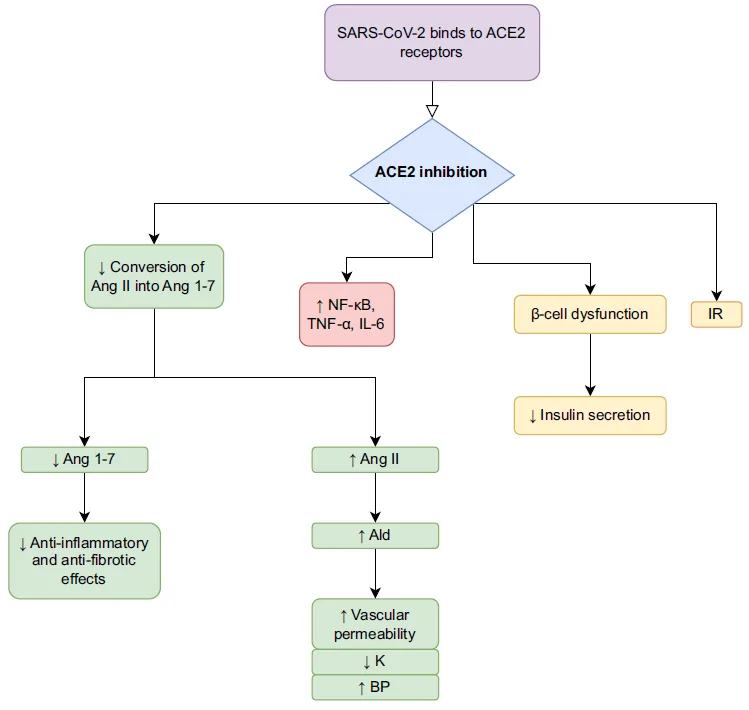
Effects of ACE2 inhibition by SARS-CoV-2 on inflammation, β-cell function, and insulin resistance. Available online under the terms of the Creative Commons Attribution Non-Commercial License 4.0. [22, 23]. ACE2, Angiotensin- converting enzyme 2; Ang II, Angiotensin II; Ang 1-7, Angiotensin 1-7; IR, Insulin resistance; BP, Blood pressure; Ald, Aldosterone; NF-κB, Nuclear factor-κB; TNF-α, Tumor necrosis factor alpha; IL-6, Interleukin 6.

**Table 1:** Temporal classification of COVID-19 illness according to the National Institute for Health and Care Excellence (NICE, 2024) based on symptom duration [13].

** COVID-19 Phase **	** Duration of COVID-1 Symptoms **
Acute COVID-19	Signs and symptoms of COVID-19 for up to 4 weeks
Ongoing Symptomatic COVID-19	Signs and symptoms of COVID-19 from 4 to 12 weeks
Post-COVID-19 Syndrome	Signs and symptoms that develop during or after an infection consistent with COVID-19, continue for more than 12 weeks, and are not explained by an alternative diagnosis

**Table 2:** Factors influencing triglyceride variability in COVID-19 research [34, 39, 41-43].

** Potential Contributors of Triglyceride Variability in COVID-19 Studies **	** Explanation / Mechanistic Rationale **
Study design differences	Retrospective studies often rely on non-standardized medical records, resulting in heterogeneous data. Prospective studies offer greater control over sample timing but often involve smaller cohorts. A meta-analysis reported only a modest increase in TG levels in severe COVID-19 cases (SMD = 0.110; 95% CI: 0.004-0.217; *p* = 0.042), with moderate heterogeneity (I ^ 2 ^ = 50.7%) [ 42 ]
Timing of sample collection	Elevated TG levels are mostly seen in the acute phase of infection, while normalization or reduction is more common during recovery. Moreover, patients with long COVID have shown secondary TG elevations [ 43 ]
Clinical and metabolic confounders	Factors such as obesity, insulin resistance, corticosteroid therapy, nutritional status, liver function, hospitalization duration, and medication regimens can all affect TG levels [34, 39].
Geographical and environmental variation	Lifestyle habits, physical activity, dietary intake, and genetic predisposition differ across populations, which may account for divergent lipid responses [41].

**Table 3:** Lipid and Glycemic Disturbances in COVID-19: Comparison between Asia and Europe [7, 8, 41, 42, 43, 60].

** Parameter **	** Asia (China, Japan, Korea) **	** Europe **
Total cholesterol (TC)	Significant decrease in severe cases	Reduced in retrospective studies
High-density lipoprotein (HDL), Low-density lipoprotein (LDL)	Reduced in severe COVID-19	Decreased across multiple studies
Triglycerides (TG)	Variable; stable or decreased; meta-analysis: increased (SMD=0.110)	Decreased in the acute phase, elevated in LCS
Haemoglobin A1C (HbA1c) (T2DM patients)	No significant changes	Worsened glycemic control

**Table 4:** Summary of the Impact of COVID-19 on Metabolism [22-29].

** Metabolic Domain **	** Patients Without T2DM **	** Patients With T2DM **	** Clinical Implications **
Total Cholesterol (TC)	Decrease (especially in severe COVID-19)	Decrease (more pronounced)	Associated with disease severity; potential prognostic marker
Low-density lipoprotein (LDL)	Decrease or stable	Decrease	Low LDL linked to poor prognosis
High-density lipoprotein (HDL)	Decrease	Decrease	Reduced immune protection; cardiovascular risk
Triglycerides (TG)	Variable: ↑ / ↓ / stable	Variable, often ↑ during the acute phase	Depends on phase, severity, and metabolic profile
Hemoglobin A1C (HbA1c)	Often stable or mildly ↑ (post-infection/lockdown)	Marked increase during the infection and pandemic period	Indicates worsened glycemic control; risk of complications
BCAAs (Leu, Ile, Val)	Decrease	Decrease	Linked to impaired energy metabolism
Uric Acid	May increase	Increase	Risk of gout and renal issues, especially in T2DM

## LIST OF ABBREVIATIONS

HDL: High-Density Lipoprotein
LDL: Low-Density Lipoprotein
Beta-CoV: Betacoronavirus
ACE2: Angiotensin-Converting Enzyme 2
PASC: Post-Acute Sequelae of COVID-19
